# Retail Cost and Energy Adjusted Cost Are Associated with Dietary Diversity and Nutrient Adequacy for Diets of 6–24 Months Children

**DOI:** 10.3390/nu14163376

**Published:** 2022-08-17

**Authors:** Tshavhuyo A. Mulabisano, Ria Laubscher, Marinel Hoffman, Jillian Hill, Ernesta Kunneke, Cornelius M. Smuts, Mieke Faber

**Affiliations:** 1 Non-Communicable Diseases Research Unit, South African Medical Research Council, Cape Town 7505, South Africa; 2 Department of Dietetics and Nutrition, University of the Western Cape, Bellville 7540, South Africa; 3 Biostatistics Unit, South African Medical Research Council, Cape Town 7505, South Africa; 4 Department of Consumer and Food Sciences, University of Pretoria, Pretoria 0002, South Africa; 5 Centre of Excellence for Nutrition (CEN), North-West University, Potchefstroom 2530, South Africa

**Keywords:** children under two, diet cost, nutrient adequacy, dietary diversity, low- and middle-income countries, South Africa

## Abstract

Poor nutrition during the first two years of life has long term consequences, but resource-poor households often do not have the means to access nutrient-dense and diverse diets. Pooled data of 24-h dietary recalls (*n* = 3336) and 2019 retail food prices were analyzed to determine associations of retail cost and energy cost (per 100 kcal) with diet quality indicators for diets of 6–24-month-old South African children who were breastfed (BF-diet) and not breastfed (NBF-diet) during the 24-h recall period. Compared to the BF-diet, retail cost for the NBF-diet was three times higher for age 6–11 months, and double for age 12–17 months. Higher retail cost and energy cost were both associated with higher mean adequacy ratios and dietary diversity scores for BF and NBF diets, except energy cost for the NBF-diet for age 6–11 months. Overall, inclusion of flesh foods, vitamin A-rich fruits and vegetables, and other fruit and vegetables increased from the lowest to the highest retail cost tertile. The higher cost of more nutritious diets highlights the importance of the affordability of diets in interventions aimed at improving diet quality. Possible strategies include: identifying the most-affordable foods within each food group, focusing on foods that provide multiple key micronutrients, and the inclusion of locally available indigenous foods.

## 1. Introduction

Children who are malnourished are at an increased risk of adverse morbidity outcomes and need a nutrient adequate and diverse diet for healthy growth and development [1]. However, many low-income households are unable to acquire a nutritionally adequate diet because of the cost of nutrient-rich foods relative to their income [2]. Low-income households who cannot afford nutritious food are prone to selecting food that is cheaper and usually of lower nutritional quality, which may result in malnutrition [3]. Most people who cannot afford a nutrient adequate diet live in Southern Asia and sub-Saharan Africa [2]. Globally in 2020, 149.2 million children younger than 5 years were stunted, 45.4 million were wasted, and 38.9 million were overweight, with most of the malnourished children living in Africa and Asia [4].

The complementary diet of children in low- and middle-income countries (LMICs) often lack key micronutrients [5], and only one in four children (aged 6–23 months) consumes a sufficiently diverse diet [6]. For 6–24-month-old children in some LIMCs, snack foods and sugar sweetened beverages provide a substantial proportion of energy intake [7]. A study in Nepal showed that children who consumed unhealthy snack foods and beverages were at higher risk of inadequate intakes for several micronutrients [8]. In a study in Asia and Africa, the majority of 6–23-month-old children were consuming commercially produced snack food and sugar-sweetened beverage products [9]. South Africa is no exception, with unhealthy foods such as sugar sweetened beverages, sugary foods, and salty snacks being introduced at a young age, and only 23% of 6–23-month-old children receiving a minimum acceptable diet [10].

Key micronutrients lacking in the complementary diet in LMICs countries are mostly iron, vitamin A, zinc, calcium, vitamin B12, and folate [11,12]. Children younger than 2 years consume small amounts of food at a time because of their small stomach capacity and therefore need nutrient-dense foods during the complementary feeding period to support growth and development [13]. The best nutrient-dense locally available food sources of multiple key micronutrients in Southern and Western Africa and South Asia include beef liver, chicken liver, small fish, eggs, and ruminant meat. Other animal source foods such as chicken, fresh milk, and fresh fish are also good sources of multiple key micronutrients but are less nutrient dense. In terms of plant foods, dark-green leafy vegetables are good sources of multiple key micronutrients, while orange vegetables and fruits such as pumpkin, carrot, and mango are rich sources of beta-carotene, which is a precursor of vitamin A [11,12]. However, it may be challenging to meet nutrient requirements, particularly for key nutrients, without including fortified foods in the complementary diet in low-income settings [14,15].

It has been reported that nutrient-dense foods cost more [16,17,18], and that energy dense but nutrient poor foods are often cheaper [17,19]. Access to sufficient nutritious foods therefore depends on cost and affordability thereof [2]. In South Africa, maize meal (which is commonly eaten as complementary food) is fortified per legislation with vitamin A, thiamine, riboflavin, niacin, pyridoxine, folic acid, iron, and zinc [20], and therefore provides a cheaper source of some of the key micronutrients.

A recently published paper reported that for diets of 6–24-month-old children in low socioeconomic settings in South Africa, underlying dietary patterns differ in diet quality based on various indicators of diet quality [21]. Using the same dataset, the aim of this study was to determine whether nutrient adequacy and dietary diversity was associated with the cost of the diet.

## 2. Materials and Methods

### 2.1. Study Design and Study Population

For the current study, an existing dataset was used which consisted of pooled data of previously collected 24-h dietary recalls (*n* = 3336) for 6–24-month-old children from four independent South African studies [21]. All four of these studies were done in resource-poor settings: two in rural sites in KwaZulu-Natal Province [22,23,24], one in both an urban and a rural area in KwaZulu-Natal Province [25], and one in a peri-urban area in North West Province [26,27].

In the two studies that were done in rural sites in KwaZulu-Natal Province [22,23,24], study participants (mother-infant pairs) for two independent randomized controlled trials (RCT) were recruited through 12 health posts of an NGO-driven community-based health program. Exclusion criteria included: premature birth (<37-week gestation), birth weight < 2500 g, hemoglobin concentration < 80 g/L [23,24], or weight-for-length z-score < −3 [24]. Dietary intake data were collected at age 6–12 months, age 12–18 months [23,24], and 18–24 months [24]. In the study that was done in a peri-urban site in North West province, study participants (mother-infant pairs) for an RCT were recruited through primary health care facilities and house-to-house visits. Exclusion criteria were: hemoglobin concentration < 70 g/L, weight-for-length z-score < −3, severe congenital abnormalities, infant known to be HIV positive, and infants known to be allergic/intolerant to peanuts, soy, cow’s milk protein, or fish. Dietary intake data were collected at age 6 months, age 12 months, and 18 months [26,27]. In the three aforementioned studies, dietary intake data were missing for children whose caregiver could not provide reliable information because the child was not in her permanent care during the 24-h recall period. The fourth study was a cross-sectional study. Primary caregivers of randomly selected children, stratified per age category (6–11 months, 12–17 months, and 18–24 months), were recruited through house-to-house visits in two study sites, one rural and one urban, in KwaZulu-Natal province [25].

The pooled dataset contained a variety of dietary intakes, ranging from mostly maize-based to substantial use of commercial infant products. As described previously [21], the 24-h dietary recalls were recoded to ensure that coding and analysis were standardized across the dietary surveys, and that all records were analyzed with the 2017 South African Food Composition Database [28], which includes an updated section on infant foods. Estimated intake of breast milk was assumed according to age: 675 mL for age 6–11 months, 615 mL for age 12–17 months, and 550 mL for age 18–24 months [29].

To determine the relationship between diet cost and diet quality indexes, the pooled dietary intake data from the four independent studies were linked to the cost of the diet using 2019 retail prices. The aim was not to determine either cost or diet quality *per se*, but rather to determine the relation between cost and diet quality over a wide range of dietary patterns, which the pooled data provided.

### 2.2. Cost of Total Intake

Retail prices for all food items reported for the 24-h recalls were obtained in 2019 from three major supermarket chains in South Africa (Pick n Pay, Checkers, and Shoprite). For each food item, average cost in South African Rands (ZAR) per 100 g was calculated, and then yield and retention factors reported by Bognár [30] were used to calculate cost per 100 g edible portion. To calculate the cost of mixed dishes, the recipes in the South African Food Quantities Manual [31] were used as a guideline. Cost per 100 g edible portion was linked to the food intake data to calculate retail cost and energy adjusted cost (cost per 100 kcal).

### 2.3. Diet Quality Indicators

Nutrient adequacy ratios (NAR) were calculated for 14 micronutrients using age-appropriate recommended dietary allowance (RDA) values or, where there is no RDA, the Adequate Intakes (AI) of the Dietary Reference Intakes (DRIs) [32,33]. To calculate the mean adequacy ratio (MAR), NARs > 1 were truncated at 1 for nutrients with intake exceeding the requirement, and the MAR was calculated as the average of the truncated NARs [34].

Nutrient density, expressed as the amount of a nutrient per 100 kcal, was calculated for 11 micronutrients, and the average nutrient density (Ave-ND) for the 11 nutrients were calculated.

The dietary diversity score (DDS) was calculated according to WHO and UNICEF guidelines [35]. The food groups that were used to calculate the DDS were: (i) Breastmilk, (ii) Grains, roots, and tubers (including infant cereal), (iii) Legumes and nuts, (iv) Dairy products (including formula milk), (v) Flesh foods (meat, poultry, organ meat, offal, and liver), (vi) Eggs, (vii) Vitamin-A rich fruits and vegetables, and (viii) Other fruits and vegetables. Commercial baby foods such as pureed foods and infant juices were categorized according to their main ingredient. If foods from a specific food group was consumed, a score of 1 was allocated to that food group, and 0 was allocated if no food item from the food group was consumed. The scores for the 8 food groups were summed to obtain the DDS, which could potentially range from 1 to 8. Minimum dietary diversity (MDD) was defined as DDS ≥ 5 [35].

### 2.4. Statistical Analysis

The data were stratified into three age categories: 6–11 months, 12–17 months, and 18–24 months. Data were further stratified within these categories, into two diet categories: whether the child was breastfed (BF-diet) or not-breastfed (NBF-diet) during the 24-h recall period.

Statistical analyses were done using STATA 16. As the data were not normally distributed, results are reported as the median (25th and 75th percentiles).

The relationship of both retail cost and energy cost with MAR, DDS, and Ave-ND respectively was determined for BF-diet and NBF-diet using Spearman correlation analysis. For each diet quality indicator (MAR, Ave-ND, DDS), median regression analysis was done with cost as dependent variable and the diet quality indictor as independent variable. The graphs are the result of using “marginsplot” after each median regression. Data were further explored by stratifying each age category according to retail cost tertiles (Ts), and then calculating the inclusion of the 8 food groups per cost tertile for BF-diet and NBF-diet respectively. A *p*-value < 0.05 was considered statistically significant.

## 3. Results

**Figure 1:**
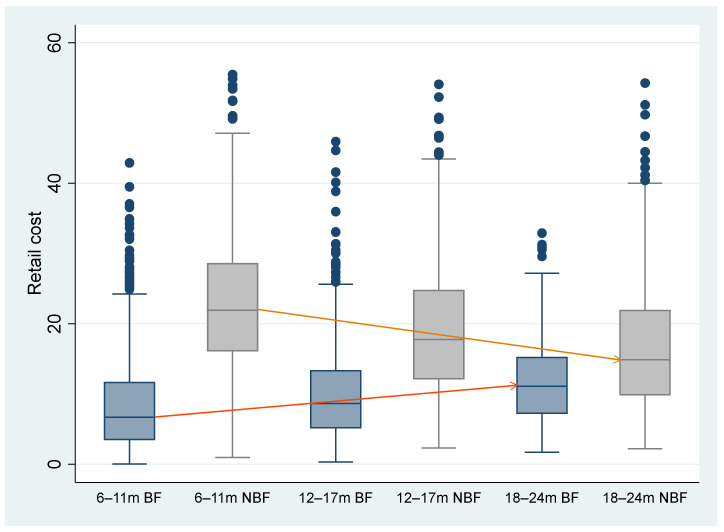
Box plots for the retail cost values per age group and diet type. BF, breastfed; m, months; NBF, non-breastfed.

Median values for diet cost and diet quality indicators are given in Table 1. Box plots for the retail cost per age group and diet type are presented in Figure 1. The difference in retail cost between the BF-diet and NBF-diet is 15.24 ZAR for 6–11 months, 9.04 ZAR for 12–17 months, and 3.77 ZAR for 18–24 months, with the NBF-diet having a higher retail cost compared to the BF-diet for all three age categories (1 ZAR = 0.059 USD). The data shows that, for the BF-diet, the retail cost is 4.4 ZAR higher for 18–24 months compared to 6–11 months, while the NBF-diet is 7.03 ZAR lower.

Food groups included in the BF and NBF diets for the three age categories are given in Table 2. Foods from the grains/roots/tubers group were almost always part of the diet. The least consumed food group was eggs, with egg being reported for only 7.3% of the 3366 24-h recalls. The inclusion of dairy in the diet increased from 42.9% at age 6–11 months to 57.8% at age 18–24 months for the BF-diet, and decreased from 95.5% to 60.3% for the NBF-diet. The dairy group includes formula milk; for the NBF-diet, formula milk was high (85.5%) at age 6–11 months compared to 13.5% at age 18–24 months. Intake of flesh foods increased across the three age categories for both BF and NBF diets. Legumes, vitamin A-rich fruits and vegetables, and other fruits and vegetables increased slightly from age 6–11 months to 12–17 months and remained fairly stable thereafter. Minimum dietary diversity was achieved for only 10.8% BF-diets and 5.4% NBF-diets at age 6–11 months. Although higher, at 18–24 months, achieving minimum dietary diversity was still low (35.7% BF-diet, 17.7% NBF-diet).

### 3.1. Associations between Cost and Diet Quality

**Figure 2:**
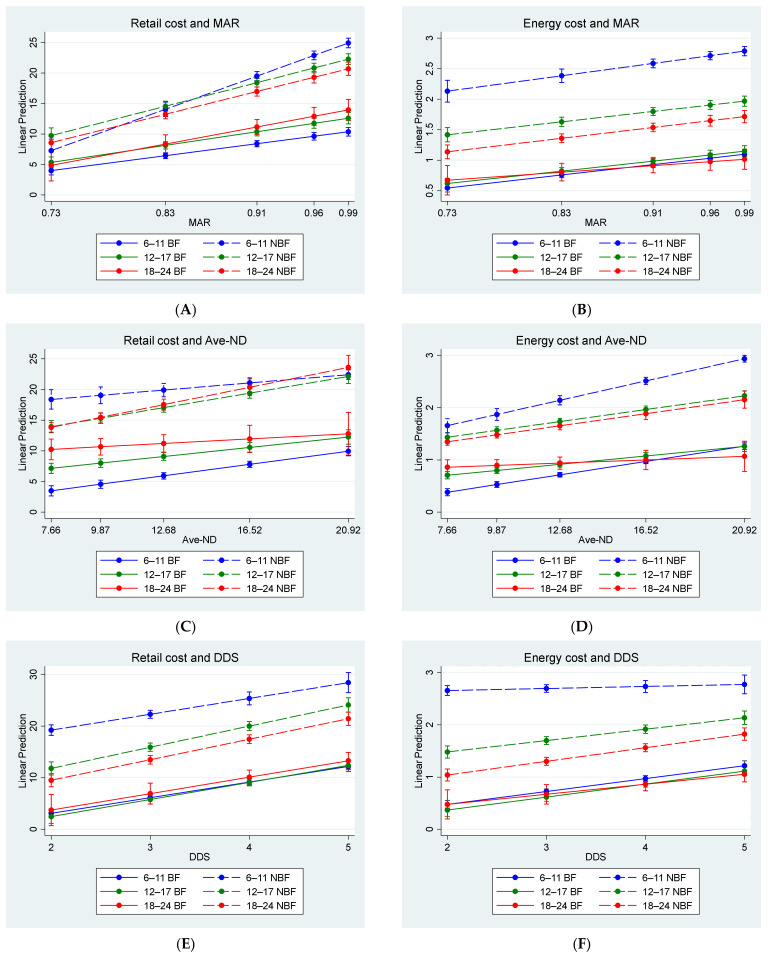
Adjusted predictions and 95% CI for retail cost and energy cost with diet quality. (**A**). Retail cost and mean adequacy ratio (MAR). (**B**). Energy cost and mean adequacy ratio (MAR). (**C**). Retail cost and average nutrient density (Ave-ND). (**D**). Energy cost and average nutrient density (Ave-ND). (**E**). Retail cost and dietary diversity score (DDS). (**F**) Energy cost and dietary diversity score (DDS).

Spearman correlation coefficients are indicated in Table 3, and the results of the median regression analysis adjusted for age-group and diet type (BF, NBF) are indicated in Figure 2A–F. Retail cost and energy cost were significantly correlated for BF and NBF diets for all three age categories. Both retail cost and energy cost were positively correlated with MAR and DDS for BF and NBF diets in all three age categories, except energy cost for the NBF-diet at 6–11 months, which did not correlate with MAR. The positive associations of cost with MAR and DDS respectively are illustrated in Figure 2. The figure further illustrates that the difference in cost between the BF-diet and NBF-diet is biggest at age 6–11 months and smallest at age 18–24 months. Positive correlations of retail cost and energy cost with Ave-ND were significant; however, the correlations were mostly weak for retail cost. Table 3 further shows that MAR and DDS were positively correlated for BF and NBF diets in all three age categories.

### 3.2. Food Groups Included in the Diet According to Cost Tertiles

**Figure 3:**
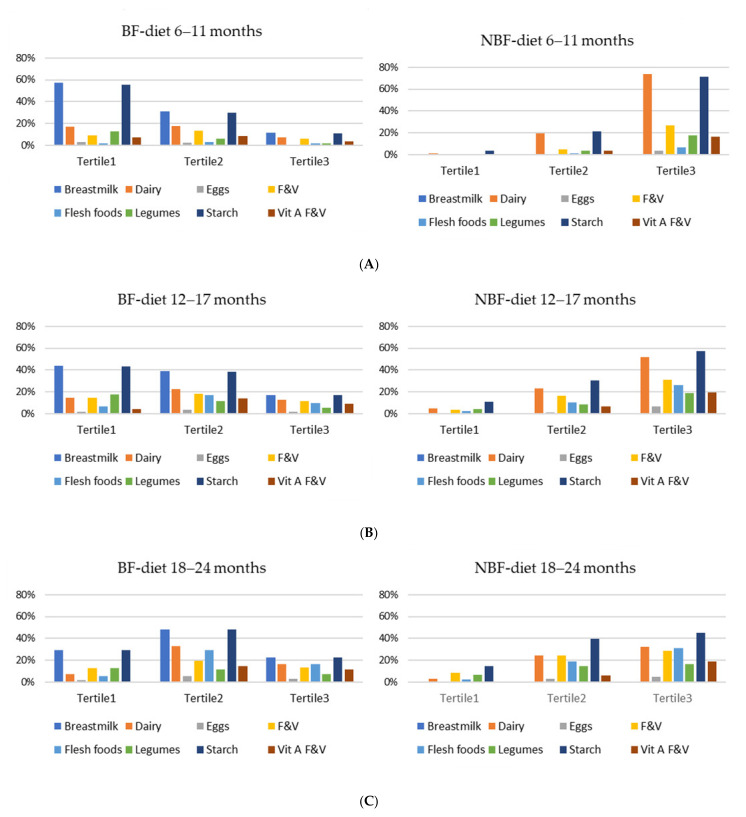
(**A**). Food groups included in the BF and NBF diets across the cost tertiles for 6–11 months. (**B**). Food groups included in the BF and NBF diets across the cost tertiles for 12–17 months. (**C**). Food groups included in the BF and NBF diets across the cost tertiles for 18–24 months. BF, breastfed; F&V, fruits and vegetables; NBF, non-breastfed.

Figure 3A–C show the food groups included in the BF-diet and NBF-diet across the retail cost tertiles for the three age categories. For 6–11 months, most of the food groups of the BF-diet fell in T1 and, to a lesser extent, in T2, while most of the food groups of the NBF-diet fell in T3. For 12–17 months, most of the food groups of the BF-diet fell in either T1 or T2, while most of the food groups of the NBF-diet fell in T3 and, to a lesser extent, T2. For 18–24 months, only a few of the food groups of the NBF-diet fell in T1.

**Figure 4:**
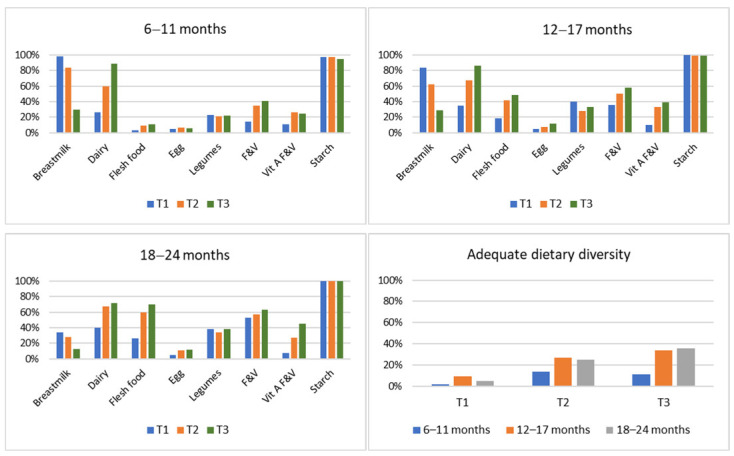
Food groups included for each of the respective retail cost tertiles and adequate dietary diversity (at least 5 of the 8 food groups) per retail cost tertile. F&V, fruits and vegetables.

For the BF and NBF diets combined, food groups included in the diet according to retail cost tertiles are shown in Figure 4. For the two younger age categories, in diets with the lowest cost (T1), at least 80% included breastmilk and <35% included dairy. On the contrary, in diets with the highest cost (T3) in these two age categories, at least 80% included dairy and approximately 30% included breastmilk. Overall, across the three age categories, the inclusion of flesh foods, other fruits and vegetables, and vitamin A-rich fruits and vegetables increased from the lowest to the highest retail cost tertile.

Table 4 shows food groups predominantly (at least 50%) included in the diet for the cost retail tertiles. The lowest cost diet (T1) predominantly consisted of cereals/root/tubers and breastmilk for the two younger age categories, and of cereals/root/tubers and fruit and vegetables (other than vitamin A-rich) for 18–24 months. Diets with the highest cost (T3) predominantly consisted of cereals/root/tubers and dairy for 6–11 months, of cereals/root/tubers, fruit and vegetables (other than vitamin A-rich), and dairy for 12–17 months, and of cereals/root/tubers, fruit and vegetables (other than vitamin A-rich), dairy, and flesh food for 18–24 months.

## 4. Discussion

Results showed that, despite the difference in retail cost between BF and NBF diets, higher dietary diversity and nutrient adequacy were associated with higher retail cost and energy cost, for both BF and NBF, except for NBF at 6–11 months for energy cost and MAR.

The retail cost of the NBF-diet was higher than that of the BF-diet for all three age categories. This result was expected as breastmilk does not have any monetary cost, which is recognized as one of the benefits of continued breastfeeding up to age 2 years and beyond [36]. Furthermore, formula milk feeds (which is part of the dairy group) probably contributed substantially to the higher cost of the NBF-diet, particularly for the two younger age categories. Compared to the BF-diet, the retail cost of the NBF-diet was three times higher at age 6–11 months and double at age 12–17 months. In 2021, the monthly food basket for a household of seven, as purchased by women living on low incomes, was estimated to cost 4039 ZAR per month, which increased by 10% to 4450 ZAR in 2022 [37]. Therefore, for low-income households, an estimated 15 ZAR per day (450 ZAR per month) difference between the BF-diet and NBF-diet at age 6–11 months is substantial. Aside from the monetary cost, not breastfeeding has substantial human and economic costs in countries with low breastfeeding rates [38].

In South Africa, where more than half of the population cannot afford a nutrient rich or healthy diet [2], breastfeeding rates are low: 51.4% at age 12 months and 13.0% at age 18 months [10]. After age 12 months, breastmilk is often not replaced with any other milk feeds and studies have shown that more than 75% of young children have an inadequate calcium intake [25,27,39]. This is of concern because 27% of children under five are stunted [10], and low intake of calcium is shown to be associated with stunting in 2–5-year-old children in a study in South Africa [40]. Milk is the main source of dietary calcium in young South African children [39], and the low intake of calcium could be contributed to insufficient consumption of milk [27,40,41]).

Only 16% of the total sample had minimum dietary diversity (at least 5 of the 8 food groups). Consuming a diet that lacks variety is common not only in infants and young children in South Africa [42], but also in adults [43]. A diet lacking diversity increases the risk of micronutrient deficiencies [44], and adequate dietary diversity has been shown to be associated with micronutrient adequacy of the complementary diet [45]. The positive associations between DDS and MAR for both BF and NBF diets in all three age categories confirm the association between dietary diversity and micronutrient adequacy.

A higher dietary diversity has been reported to be associated with higher nutrient density for protein and several micronutrients of the complementary diet for 12–24-month-old children in South Africa [25]; however, the results showed no strong correlation between DDS and Ave-ND. At age 6–11 months, the diet predominantly consisted of either breastmilk or formula milk, and foods from the grains/root/tubers group, which includes both maize meal and infant cereal. The DDS indicator does not distinguish between fortified and unfortified foods [35]; therefore, the lack of any strong correlation between DDS and Ave-ND can most likely be attributed to fortified maize meal and fortified infant cereals both being included in the grains/roots/tuber group.

The positive association of retail cost with both MAR and DDS indicate that a more nutritious and diverse diet will cost more. Globally, nutritious food such as fruits, vegetables, and flesh foods typically cost more than foods with high energy density but low nutrient content [46]. Providing children with diverse diets that are nutrient-dense and nutritionally adequate is challenging in low-income households, particularly in eastern and southern African countries, where households generally rely primarily on low cost, nutrient poor staples [3]. In South Africa, cost is the most important factor influencing food choices [47,48]. Nutrient profiling can be used to identify the most affordable nutrient-dense foods within each food group [49], which may assist consumers in making healthy food choices at the lowest cost and while also increasing dietary diversity. Furthermore, focusing on locally available food sources of multiple key nutrients may reduce the cost of a nutritionally adequate diet [11,12].

The consumption of flesh foods in children 6 to 23 months of age in South Africa is generally low [42]. In the current study, inclusion of flesh food in the diet was highest in the highest cost tertile. Animal source foods are typically more expensive in low-income countries [17], and, in LIMICs, consumption of animal source foods is low due to poor accessibility and affordability [36,50]. Egg, which was the least consumed food-group for all three age categories, is a one of the cheapest animal source foods in South Africa [51] and mothers should be encouraged to give their infants and small children eggs more frequently; legumes are also a good source of protein and key micronutrients. Inclusion of legumes/nuts did not differ across the diet cost tertiles. Legumes (beans) is one of the core foods (prioritized and bought first) in the food basket of low-income households in South Africa [37].

Vitamin A-rich fruits and vegetables were the second least consumed food group, and inclusion in the diet was highest in the highest cost tertile. Cost is a limiting factor for consumption of fruits and vegetables in resource poor settings [52], and achieving the revised pediatrics food-based dietary guideline that suggests that children aged 12 to 36 months should have vitamin A-rich fruits and vegetables (dark-green leafy vegetables and orange-colored vegetables and fruits) daily [53] will be difficult. It has been suggested that consumption of indigenous foods could potentially improve micronutrient intakes in infants and young children [54]. Promoting indigenous vegetables and fruits as a complementary food could be a low-cost strategy to improve dietary diversity and micronutrient intake of children, particularly in rural areas in South Africa.

The results of the study should be interpreted within the inherent limitations of dietary assessment methodology [55]. Furthermore, quantifying breastmilk intake is challenging, and the nutrient content of breastmilk may vary depending on the mother’s dietary intake and nutritional status [56]. In the current study, age-specific average breastmilk intake values [29] and average nutrient content values for breastmilk [28] were used. The time interval between the original studies and the time lapse since data collection should have little impact on the results of this study, as all 24-h recalls in the dataset were standardized in coding and nutrient analysis, and the cost of all dietary intakes was determined using retail prices at a specific time. The focus of this paper is to ascertain the associations of diet cost and diet quality, not to report actual diet costs. Due to inflation and rising food costs, the actual cost of the diet will differ over time, but it is postulated that the association between cost and diet quality will most likely show the same trends as found in the current study.

## 5. Conclusions

The higher cost of more nutritious diets, as observed in the current study, highlights the importance of considering affordability of the diet in interventions aimed at improving diet quality in children under 2 years of age. The higher cost of the NBF diet is a financial strain for low-income households and could be used as deterrent for not breastfeeding. Dietary diversity was low for both the BF and NBF diets. Possible strategies to improve dietary diversity include identifying the most affordable foods within each food group, focusing on foods that provide multiple key micronutrients, and the inclusion of locally available indigenous foods.

## Figures and Tables

**Table 1:** Median (P25, P75) for cost and diet quality indicators per age group and type of diet.

	6–11 Months		12–17 Months		18–24 Months	
	BF-Diet	NBF-Diet	BF-Diet	NBF-Diet	BF-Diet	NBF-Diet
Sample size	1119	466	658	473	154	466
Retail cost (ZAR)	6.68	21.92	8.66	17.77	11.12	14.89
	(3.42, 11.75)	(16.06, 28.67)	(5.08, 13.43)	(12.05, 24.8)	(7.14, 15.31)	(9.79, 21.99)
Energy cost (ZAR/100 kcal)	0.82	2.70	0.88	1.80	0.92	1.45
	(0.46, 1.34)	(2.10, 3.32)	(0.56, 1.27)	(1.37, 2.31)	(0.70, 1.20)	(1.03, 1.98)
MAR	0.88	0.97	0.88	0.94	0.92	0.89
	(0.80, 0.94)	(0.93, 1.00)	(0.80, 0.94)	(0.86, 0.98)	(0.86, 0.97)	(0.82, 0.95)
Ave-ND	13.88	18.23	11.05	12.48	10.03	8.39
	(11.63, 16.60)	(15.25, 21.85)	(9.54, 13.79)	(9.01, 16.42)	(8.99, 11.80)	(6.38, 11.08)
DDS	3.0	3.0	4.0	3.0	4.0	3.0
	(3.0, 4.0)	(2.0, 3.0)	(3.0, 5.0)	(3.0, 4.0)	(3.0, 5.0)	(3.0, 4.0)

Ave-ND, average nutrient density; BF, breastfed; DDS, dietary diversity score; MAR, mean adequacy ratio; NBF, non-breastfed; ZAR, South African Rand; 1 ZAR = 0.059 USD.

**Table 2:** Food groups included in BF and NBF diets for the three age categories, *n* (%).

	6–11 Months		12–17 Months		18–24 Months	
	BF-Diet	NBF-Diet	BF-Diet	NBF-Diet	BF-Diet	NBF-Diet
Sample size	1119	466	658	473	154	466
Breastmilk	1119 (100)	0 (0)	658 (100)	0 (0)	154 (100)	0 (0)
Grains, roots and tubers	1083 (96.8)	451 (96.8)	655 (99.7)	470 (99.4)	154 (100)	466 (100)
Legumes	243 (21.7)	102 (21.9)	232 (35.3)	152 (32.1)	50 (32.5)	176 (37.8)
Dairy (incl. formula milk)	480 (42.9)	445 (95.5)	332 (50.5)	375 (79.9)	89 (57.8)	281 (60.3)
Formula milk	275 (24.6)	400 (85.8)	70 (10.6)	207 (43.8)	5 (3.2)	63 (13.5)
Flesh foods ^1^	87 (7.8)	40 (8.6)	228 (34.7)	118 (39.7)	80 (51.9)	244 (52.4)
Eggs	70 (6.3)	23 (4.9)	51 (7.8)	42 (8.9)	17 (11.0)	41 (8.8)
Vit A-rich fruits & vegetables	277 (20.3)	96 (20.6)	185 (28.1)	127 (26.8)	42 (27.3)	124 (26.6)
Other fruits & vegetables	326 (29.1)	150 (32.2)	297 (45.1)	246 (52.0)	71 (46.1)	286 (61.4)
Minimum DD (≥5 groups)	121 (10.8)	25 (5.4)	198 (30.1)	66 (14.0)	55 (35.7)	82 (17.6)

BF, breastfed; DD, dietary diversity; NBF, non-breastfed; ^1^ meat, poultry, organ meats, offal & liver.

**Table 3:** Spearman correlation coefficients for diet cost and diet quality.

		6–11 Months		12–17 Months		18–24 Months	
		BF-Diet	NBF-Diet	BF-Diet	NBF-Diet	BF-Diet	NBF-Diet
Sample size		1119	466	658	473	154	466
Retail cost versus energy cost	*r*	0.961	0.550	0.933	0.748	0.870	0.848
	*p*-value	<0.001	<0.001	<0.001	<0.001	<0.001	<0.001
Retail cost versus MAR	*r*	0.610	0.680	0.671	0.732	0.664	0.682
	*p*-value	<0.001	<0.001	<0.001	<0.001	<0.001	<0.001
Retail cost versus Ave-ND	*r*	0.311	0.175	0.283	0.464	0.181	0.417
	*p*-value	<0.001	<0.001	<0.001	<0.001	0.025	<0.001
Retail cost versus DDS	*r*	0.516	0.279	0.606	0.415	0.575	0.500
	*p*-value	<0.001	<0.001	<0.001	<0.001	<0.001	<0.001
Energy costs versus MAR	*r*	0.436	0.028	0.466	0.387	0.343	0.422
	*p*-value	<0.001	0.542	<0.001	<0.001	<0.001	<0.001
Energy cost versus Ave-ND	*r*	0.409	0.523	0.401	0.601	0.200	0.417
	*p*-value	<0.001	<0.001	<0.001	<0.001	0.013	<0.001
Energy cost versus DDS	*r*	0.441	0.104	0.566	0.298	0.598	0.452
	*p*-value	<0.001	0.025	<0.001	<0.001	<0.001	<0.001
DDS versus MAR	*r*	0.515	0.243	0.478	0.306	0.356	0.462
	*p*-value	<0.001	<0.001	<0.001	<0.001	<0.001	<0.001
MAR versus Ave-ND	*r*	0.137	0.077	0.211	0.538	0.335	0.646
	*p*-value	<0.001	0.096	<0.001	<0.001	<0.001	<0.001
DDS versus Ave-ND	*r*	−0.045	−0.185	0.091	0.094	0.122	0.327
	*p*-value	0.137	<0.001	0.020	0.040	0.132	<0.001

Ave-ND, average nutrient density; BF, breastfed; DD, dietary diversity; MAR, mean adequacy ratio; NBF, non-breastfed.

**Table 4:** Food groups predominantly included (>50%) according to retail cost tertiles.

Age Category	Retail Cost Tertiles		
	Tertile 1	Tertile 2	Tertile 3
6–11 months	Grains/roots/tubers Breastmilk	Grains/roots/tubers Breastmilk Dairy	Grains/roots/tubers Dairy
12–17 months	Starchy foods Breastmilk	Starchy foods Breastmilk Dairy Fruit and vegetables	Starchy foods Dairy Fruit and vegetables
18–24 months	Starchy foods Fruit and vegetables	Starchy foods Fruit and vegetables Dairy Flesh food	Starchy foods Fruit and vegetables Dairy Flesh food

## Data Availability

Permission to obtain the data can be request from the corresponding author.
